# Combined intracoronary assessment and treatment of a patient with coronary plaque rapid progression prior to acute myocardial infarction: A case report

**DOI:** 10.1097/MD.0000000000015621

**Published:** 2019-05-13

**Authors:** Daoyuan Si, Beibei Du, Yanan Zhao, Xiangdong Li, Xingtong Wang, Kun Liu, Yuquan He, Ping Yang

**Affiliations:** a Jilin Provincial Engineering Laboratory for Endothelial Function and Genetic Diagnosis of Cardiovascular Disease, Department of Cardiology, China-Japan Union Hospital of Jilin University; b Department of Oncology and Hematology, Cancer Center, The First Hospital of Jilin University, Changchun, Jilin, China.

**Keywords:** acute myocardial infarction, mild stenosis, optical coherence tomography, plaque erosion, rapid progress

## Abstract

**Rational:**

Plaque rapid progression prior to acute myocardial infarction is not a common phenomenon, and its mechanism remains unknown. Intracoronary imaging may help to assess the plaque characteristics and progression.

**Patient concern:**

A 37-year-old male patient suffered an acute myocardial infarction (AMI) 1 month after the diagnosis of a mildly stenosed coronary artery. Intracoronary imaging was done to seek the underlying causes and guide further treatment.

**Diagnosis:**

Two coronary angiograms in 1 month showed plaque rapid progressing prior to the AMI. Intracoronary optical coherence tomography (OCT) post-AMI showed plaque erosion and heavy burden of thrombus.

**Intervention:**

The patient was advised to defer stent deployment. The patient was then given intensified antithrombotic therapy. Three weeks later, OCT imaging revealed sufficient lumen area and the intact endothelium without remaining thrombus. Fractional flow reverse (FFR) showed no functional ischemia. Dual-antiplatelet therapy without stenting was recommended for 12 months.

**Outcomes:**

The 6-month follow-up showed good recovery and normal cardiac function.

**Lessons:**

First, for patients with mild coronary stenosis and typical angina symptoms, further intracoronary assessment should be performed. Second, OCT can not only help to determine the plaque characteristics but can also help to develop patient-tailored strategies for AMI patients.

## 1 Introduction

Acute myocardial infarction (AMI) is often caused by acute coronary occlusion or severe stenosis. However, the preinfarction culprit lesion can be only mild stenosis. Some mildly stenotic plaques appear to progress rapidly to severe stenosis or total occlusion, possibly causing acute coronary syndrome.^[1]^ To date, the mechanisms of rapid progression of plaque prior to acute myocardial infarction remain unclarified. The most commonly used method for assessing stenosis and plaque burden is visual evaluation or quantitative coronary arteriography (QCA) assessment by coronary angiography. However, coronary angiography provides limited information and often neglects patients with mild stenosis but with vulnerable plaque.^[2]^ Such patients can quickly progress to acute myocardial infarction. Here, we report the interventional process and intracoronary assessment of an AMI patient with angiographic mild stenosis at baseline 1 month prior.

## 2 Case presentation

**Figure 1:**
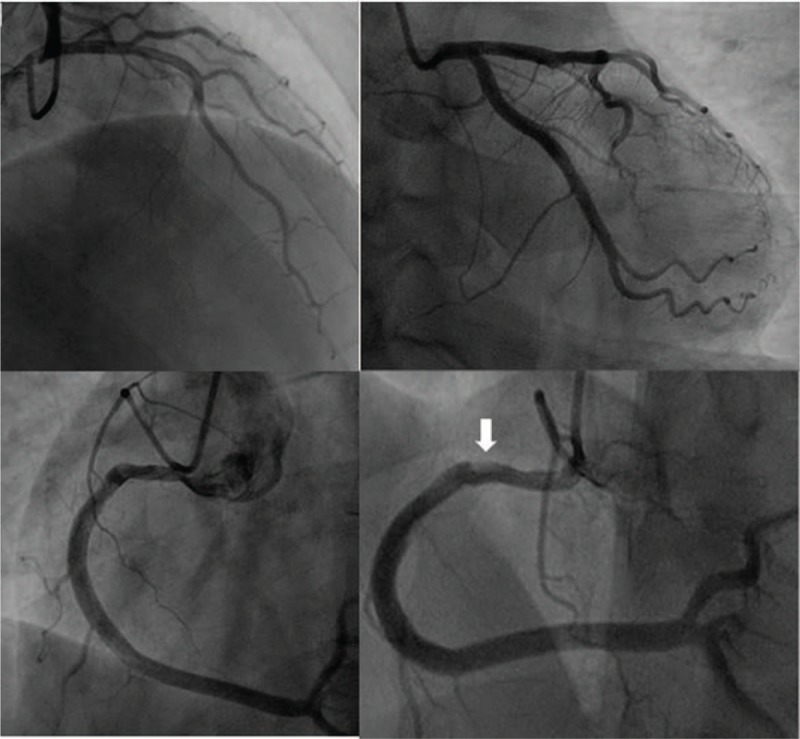
Baseline angiogram. There is mild stenosis in proximal RCA. A filling defect in the proximal RCA (arrow). RCA = right coronary artery.

A 37-year-old male patient was hospitalized for paroxysmal chest pain for 1 week with aggravation upon exercise in another hospital. Electrocardiography (ECG) showed no significant ischemic changes. Myocardial biomarkers were normal. Selective coronary angiography (CAG) showed only mild stenosis in the proximal segment of the right coronary (RCA) artery (see Fig. 1). QCA was performed to check the severity of the stenosis. The mean diameter stenosis by QCA was 28%, and the mean area stenosis by QCA was 42%. The patient was told that the coronary arteries were basically normal, and the pain was not related to the heart. The patient was also advised to discontinue the antiplatelet drugs.

**Figure 2:**
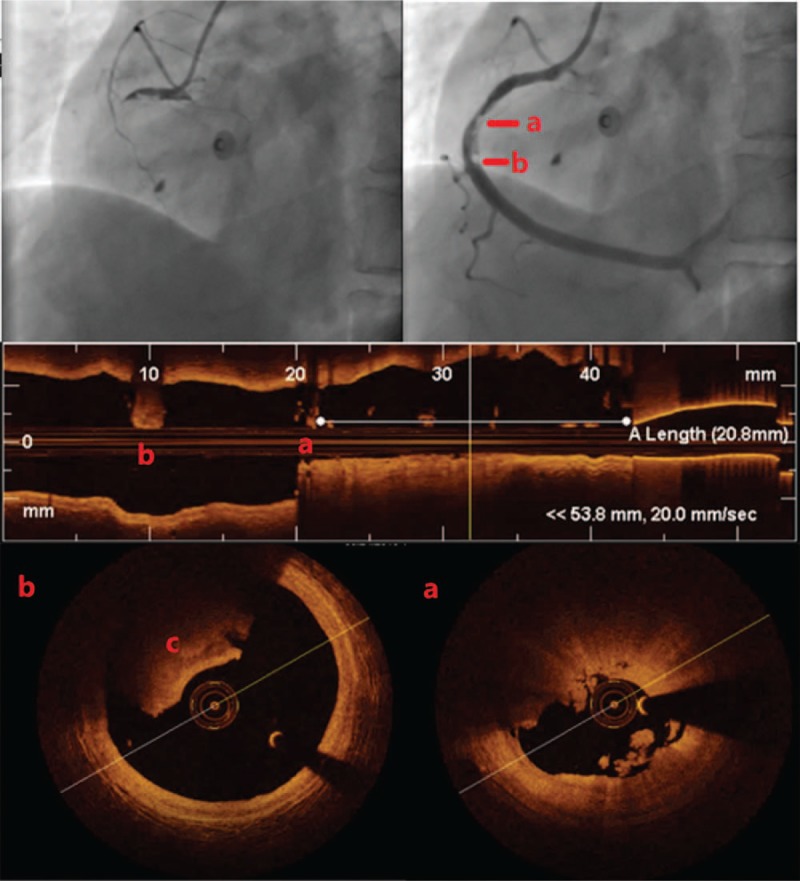
Coronary angiogram before and after thrombus aspiration, longitudinal OCT, and cross-sectional OCT images. A, The lesion 20.8 mm to the ostium of RCA, red thrombus (9:00–2:00 o’clock) and white thrombus (5:00–7:00 o’clock). The minimum lumen area, 5.86 mm^2^. B, The residue thrombus at 32 mm to the ostium of RCA, red thrombus at 9:00–1:00 o’clock. C, Red thrombus, medium reflectivity, and high attenuation. OCT = optical coherence tomography, RCA = right coronary artery.

**Figure 3:**
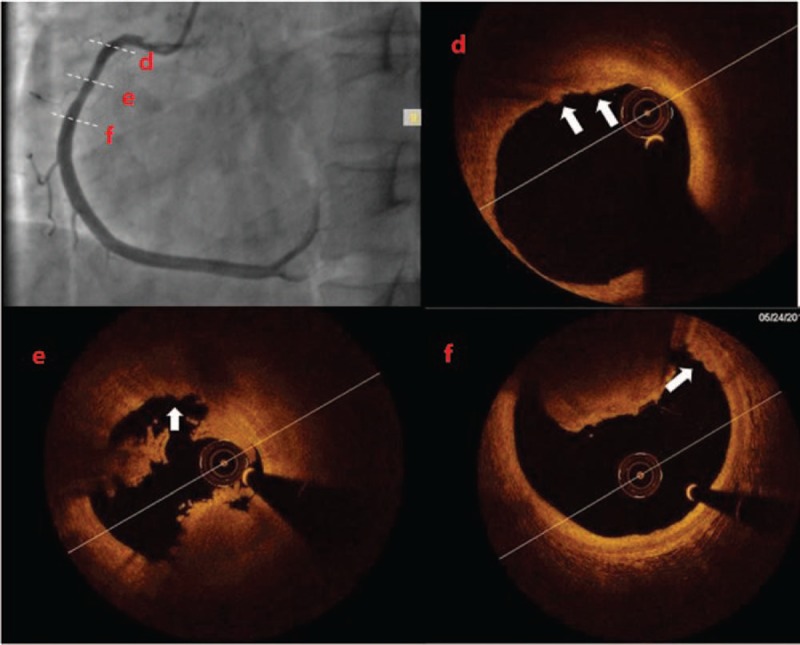
Representative OCT-erosion cross-sectional changes in different segments of the RCA (D–F, arrows). OCT = optical coherence tomography, RCA = right coronary artery.

One month later, the patient was admitted to our hospital on an emergency basis because of persistent chest pain, lasting approximately 1 hour. ECG showed ST-segment elevation in the inferior wall lead, TnI 0.8 ng/mL. Coronary angiography showed acute occlusion of the right coronary artery. A 6F JR4.0 guiding catheter (Cordis, UA) was engaged, and a Runthrough NS guidewire (Terumo, JP) easily passed the occlusion. After repeated thrombus aspiration (EXPORT AP, Medtronic, UA), TIMI flow returned to grade 3; however, there remained residual thrombus in the middle segment of the RCA (see Fig. 2). Optical coherence tomography (OCT) was checked to determine the etiology of the myocardial infarction and to measure the residual lumen area. As a result of the presence of residual red thrombus, the underlying structure was not well visualized in the corresponding OCT images. Nevertheless, there were representative OCT-erosion changes in various segments of the RCA (see Fig. 3). Furthermore, there was no detectable plaque rupture or cavity formation. The minimum lumen area (MLA) of the middle RCA was 5.86 mm^2^, and the area stenosis (AS) percentage was 65% (see Fig. 2). As plaque erosion was the etiology of the infarction, the residual diameter stenosis (DS) was <70% on angiogram, TIMI flow grade was 3, and the patient was stable without symptoms. The patient was advised to defer stent deployment. With the presence of heavy burden of residual thrombus, this can help to prevent no-flow phenomenon. The patient was then given intensified antithrombotic therapy (aspirin 100 mg QD, ticagrelor 90 mg BID, and GPI for 24 hours) postprocedure. Additional echocardiograms were checked to rule out embolism.

**Figure 4:**
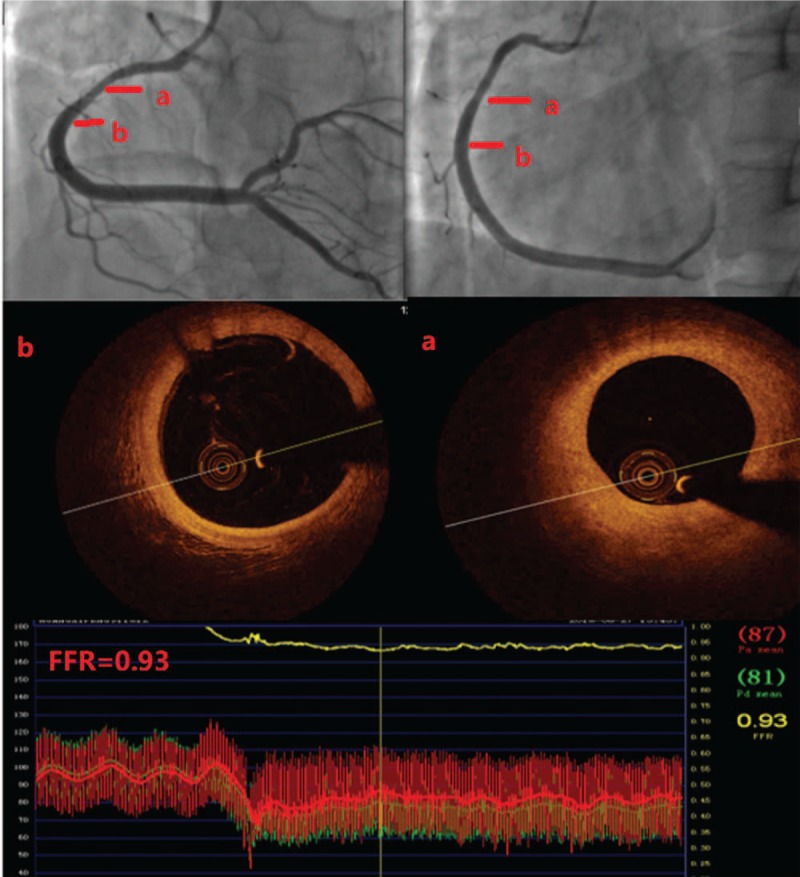
The CAG, OCT, and FFR of the RCA. A, The lesion 20.8 mm to the ostium of RCA, no thrombus left. Lumen area=7.42 mm^2^. B, The segment 32 mm to the ostium of RCA, no thrombus left. FFR=0.93. CAG = coronary angiography, FFR = fractional flow reverse, OCT = optical coherence tomography, RCA = right coronary artery.

After 3 weeks, CAG and OCT were checked again, showing that the thrombus completely disappeared, and the endothelium was intact in the previous culprit lesion segment, confirming plaque erosion as the reason for the AMI. The lumen was large enough with only mild to moderate stenosis (MLA= 7.42 mm^2^ AS% = 49.2%), and there were no vulnerable plaques. The patient avoided stenting exclusively with medication. To guarantee the safety of the patient, fractional flow reverse (FFR) for the RCA was 0.93, meaning no functional ischemia (see Fig. 4). The patient was advised to undergo 12-month dual antithrombotic and lipid-lowering therapy. At the current half-year follow-up, there were no symptoms such as chest pain, angina or chest tightness, and echocardiography showed normal cardiac function.

## 3 Discussion

The progression of coronary plaque is a quite complex process that is influenced by many factors. There have been many studies focused on the detection of coronary plaque progression; however, few studies^[3,4]^ have been conducted regarding its mechanisms. Two studies^[4,5]^ confirmed that a small portion of patients have rapid progression from mild stenosis to severe stenosis within a few months. Rapid progression of coronary stenosis to acute myocardial infarction is considered a dangerous situation.^[6]^ This is because the mildly or moderately stenosed lesions at baseline are often ignored by patients and even their doctors, and there is no necessary further examination and treatment. In the early stage of plaque progression, progression expands outward toward the media and adventitia, and the coronary artery is positively remodeled. As a result, the angiogram only shows mild stenosis compared with normal segments. However, this “mild” stenosis tends to represent thin-cap fibroatheromas or a large plaque burden on intracoronary evaluation.^[4,7]^

Mild stenosis of the right coronary in this case at baseline was quite deceptive, and as a result, the patient was advised to discontinue antiplatelet agents, eventually leading to an adverse cardiovascular event. The preinfarction “culprit lesion” may have been an eccentric focal vulnerable plaque in the first turn of the RCA (see Fig. 1, arrow) where a filling defect can be found only if seen carefully. Furthermore, if the patient had further intravascular evaluations at baseline after CAG considering his typical symptoms, the event may have been avoided. Therefore, as a lesson, if a patient has mild or moderate stenosis but with typical angina pectoris, intracoronary imaging (such as intravascular ultrasound or OCT imaging) should be routinely used to determine plaque properties and plaque burden.

Intracoronary imaging can not only help interventional cardiologists to determine the plaque characteristics but can also help to develop patient-tailored strategies for AMI patients. For this case, the deferred stenting strategy is the combined consideration of preventing no-flow phenomenon and possibly avoiding stenting with evaluation afterwards.

The no-flow or slow-flow phenomenon is common in AMI patients with high thrombus burden. As a choice of treatment for these patients, deferring stenting in primary PCI can reduce no-reflow and may increase myocardial salvage.^[8]^ Despite repeated thrombus aspiration, the existence of heavy burden of residual thrombus in the proximal RCA can result in high incidence of no-flow during subsequent dilation or stenting. No-flow in the RCA can cause severe angina or life-threating arrhythmias as the vast blood supply area of the dominant RCA. Furthermore, an erosion study^[9]^ demonstrated that if the culprit lesion shows plaque erosion confirmed by OCT imaging and if the residue diameter stenosis is <70%, TIMI flow grade is 3, and the patient is stable without symptoms, antithrombotic therapy without stenting is a possible choice. For the primary intervention for the AMI in this case, the patient is a good candidate for erosion study and meets all the above-mentioned requirements; therefore, we chose to avoid immediate stenting.

For subsequent treatment, we performed angiogram, OCT and FFR 3 weeks after the myocardial infarction to guarantee the safety of the patient. The angiogram and OCT confirmed plaque erosion and showed no residual thrombus left with sufficient lumen area in the RCA. FFR showed no functional ischemia. As a result, the patient was treated with antithrombotic treatment without stenting. Six-month follow-up also verified a good clinical outcome. The treatment for AMI can vary with the help of OCT, easily determining the pathology of the infarction,^[10]^ the burden of the thrombus, and the minimum area of the culprit vessel. For follow-up, OCT can show a clear picture of intima recovery and can check whether residual thrombus is present.

## 4 Conclusion

Coronary plaque progression is not a linear, consistent process. Some patients may experience rapid progression of coronary plaque. For patients with mild coronary stenosis but with typical angina symptoms, further intracoronary assessment should be performed. Currently, the mechanism of rapid plaque progression remains unclear, and more attention should be paid to it. In patients with acute myocardial infarction with OCT-confirmed plaque erosion, no residual thrombus, sufficient luminal area, and normal FFR result can guarantee good prognosis for avoiding stent implantation.

## Author contributions

**Conceptualization:** Daoyuan Si, Ping Yang.

**Funding acquisition:** Daoyuan Si.

**Investigation:** Beibei Du, Yanan Zhao, Xiangdong Li, Kun Liu.

**Methodology:** Xiangdong Li, Kun Liu.

**Project administration:** Xingtong Wang.

**Supervision:** Yuquan He, Ping Yang.

**Writing – original draft:** Daoyuan Si, Beibei Du.

**Writing – review & editing:** Ping Yang.

Daoyuan Si orcid: 0000-0003-3493-2300.
